# Exploring Non-Attendance Rates in the Tri-Borough Perinatal Service: An Audit of Demographic and Socioeconomic Predictors

**DOI:** 10.1192/bjo.2025.10614

**Published:** 2025-06-20

**Authors:** Precious Jolugbo, Jamila Carey

**Affiliations:** 1 Imperial College London, London, United Kingdom; 2 West London NHS Trust, London, United Kingdom

## Abstract

**Aims:** Non-attendance at initial assessment appointments in perinatal services can delay crucial care and negatively affect maternal and infant health outcomes. The Tri-Borough Perinatal Service, covering Ealing, Hounslow, and Hammersmith and Fulham, aims to maintain a non-attendance (DNA) rate of 15% or lower. This study assessed DNA rates for initial assessments and explored demographic and socioeconomic factors to identify potential predictors of non-attendance.

**Methods:** Retrospective data from 369 patients scheduled for initial assessments between August and October 2024 in the Tri-Borough Perinatal Service were analysed. After excluding duplicates and incorrectly labelled DNAs, 283 patient records remained. Demographic variables considered included age, ethnicity, self-referral status, need for a translator, disability status, and receipt of benefits. Socioeconomic deprivation was assessed using the Index of Multiple Deprivation (IMD) Rank, based on the English Indices of Deprivation 2019. Statistical analyses, including Chi-square test and binary logistic regression, were conducted to identify significant associations between these factors and DNA rates. A p-value of <0.05 was considered statistically significant.

**Results:** The overall DNA rate for initial assessments was 35.3% (n=100), which exceeds the gold standard. The average age of patients was 30 years. Most patients (94.7%) were referred by an external body (e.g. midwife, GP, health visitor), 16.6% required a translator, and 15.2% had a known disability. 101 patients (35%) were recorded as receiving benefits, although this was not recorded for 30 patients (10.6%). Ethnicity was not significantly related to DNA rates (p=0.062), with White British patients comprising 16.3% (n=46) of the sample, however 18% (n=51) of ethnicity data was missing due to not being recorded. DNA rates were significantly affected by appointment location (p=0.035), with the highest rates observed for physical centre appointments (40.0%), compared with home visits (20.7%) and remote appointments (25.9%). Socioeconomic deprivation, as measured by the IMD Rank, was a strong predictor of DNA rates (p<0.001), with higher deprivation correlating with higher non-attendance.

**Conclusion:** Socioeconomic deprivation and appointment location were found to be key factors influencing non-attendance, with higher DNA rates observed in more deprived areas and for physical centre appointments. These findings suggest that further improvement studies will be necessary to explore interventions such as alternative appointment formats and targeted support for patients from disadvantaged backgrounds, which may help reduce non-attendance and improve engagement with the service.

